# Self-Reported Depression Is Associated With Aberration in Emotional Reactivity and Emotional Concept Coding

**DOI:** 10.3389/fpsyg.2022.814234

**Published:** 2022-06-24

**Authors:** Himansh Sheoran, Priyanka Srivastava

**Affiliations:** Perception and Cognition Research Group, Cognitive Science Lab, Kohli Center on Intelligent Systems, International Institute of Information Technology, Hyderabad, India

**Keywords:** risks of or vulnerability to depression, emotional reactivity, property generation task, affective/emotion conceptual representation, semantic analysis, emotion polarity analysis

## Abstract

Cognitive impairment, alterations in mood, emotion dysregulation are just a few of the consequences of depression. Despite depression being reported as the most common mental disorder worldwide, examining depression or risks of depression is still challenging. Emotional reactivity has been observed to predict the risk of depression, but the results have been mixed for negative emotional reactivity (NER). To better understand the emotional response conflict, we asked our participants to describe their feeling in meaningful sentences alongside reporting their reactions to the emotionally evocative words. We presented a word on the screen and asked participants to perform two tasks, rate their feeling after reading the word using the self-assessment manikin (SAM) scale, and describe their feeling using the property generation task. The emotional content was analyzed using a novel machine-learning algorithm approach. We performed these two tasks in blocks and randomized their order across participants. Beck Depression Inventory (BDI) was used to categorize participants into self-reported non-depressed (ND) and depressed (D) groups. Compared to the ND, the D group reported reduced positive emotional reactivity when presented with extremely pleasant words regardless of their arousal levels. However, no significant difference was observed between the D and ND groups for negative emotional reactivity. In contrast, we observed increased sadness and inclination toward low negative context from descriptive content by the D compared to the ND group. The positive content analyses showed mixed results. The contrasting results between the emotional reactivity and emotional content analyses demand further examination between cohorts of self-reported depressive symptoms, no-symptoms, and MDD patients to better examine the risks of depression and help design early interventions.

## Introduction

Persistent sad mood and/or loss of interest or pleasure in normal daily activities along with subsidiary symptoms of significant weight loss, insomnia, inappropriate guilt and fatigue are generally regarded as the main symptoms of Major Depressive Disorder [MDD] (5th ed.; American Psychiatric Associations [APA], 2013). Depression impairs the ability to manage everyday activities and significantly contributes to suicide deaths. A recent study (Santomauro et al., 2021) on the global prevalence and burden of depressive disorder, covering 204 countries and territories, reports that MDD affects every 3152.9 cases per 100,000 population, equivalent to 246 million people, after adjusting for the COVID-19 pandemic. Using Global Burden of Disease, Injury, and Risk Factor Study data, Ogbo et al. (2018) reported that India, among the five South Asian countries, generates the most significant number of disability-adjusted life years (DALYs), 577.8 per 100,000 population because of depressive disorders and MDD. Another population-based study, specific to the south Indian region, reported the prevalence of depression to be 15.1% in south Indian states and cities (Poongothai et al., 2009). Interesting enough, the numbers of the ground are assumed to be even higher (Kohn et al., 2004).

Depression or clinically defined uni-polar depression is characterized as a mood or affective disorder associated with ineffective emotion regulation. Often mood and emotion are used interchangeably when in reality, they are different constructs. *Emotion* is defined as “a complex reaction pattern, involving experiential, behavioral, and physiological elements” (5th ed.; American Psychiatric Associations [APA], 2013). Emotional experiences are more intense and get influenced by a specific event, object, or situation (Kaplan et al., 2016). In contrast, the mood is considered a general affective state, such as feeling cheerful and influenced by a wider range of events or situations than a specific event, situation, or object. Mood persists longer than emotional experiences (Beedie et al., 2005; Kaplan et al., 2016). The current manuscript examines the role of mood in emotional reaction and emotional concept representation. We begin by discussing the relevant literature on emotional reactivity (section “Depression and Emotion”), and emotional concept representation (section “Emotion and Concept Representation”), and then describe the importance of the present study (section “Present Study”).

### Depression and Emotion

Increased levels of negative affect and decreased positive affect are associated with the depressive state or mood. However, the theoretical models of emotional dysfunction in depression present differing views. Beck’s cognitive model of depression (Beck et al., 1979), commonly known as *Cognitive Triad*, argues that an individual’s internal representation determines how an individual would perceive themselves and view the future and the world around them. During depressive episodes, individuals tend to interpret and perceive situations and things around them pessimistically (Beck et al., 1979) suggesting mood-congruent acquisition and processing of information. For example, a negative schema comprising themes of loss, failure, worthlessness, and rejection leads to increased emotional reactivity for negative *versus* positive stimuli (Farb et al., 2012). *Emotional reactivity* is defined as the tendency to experience recurrent and intense emotional arousal, which is measured as subjective, behavioral or physiological changes to an emotionally evocative stimulus.

However, contrary to the mood-congruent argument, the emotional context insensitivity (ECI) presents an integrated view of negative and positive emotional reactivity (NER and PER) to negative and positive emotional stimuli, respectively. The ECI suggests reduced reactivity to external stimuli, i.e., negative and positive attenuation, regardless of its affective nature (Bylsma et al., 2008; Benning and Ait Oumeziane, 2017). The proponents of the theory of ECI (Rottenberg, 2017) argue that depression overrides normal emotional functioning, invokes reduction in motivation, and promotes an internal focus. During depressive episodes, the individual demonstrates resistance to the emotional fluctuation representing emotional inertia and its association with psychological adjustment (Kuppens et al., 2010; Kahn et al., 2019). Less sudden changes, pervasive states and diminished responsiveness to both positive and negative emotions are a few characteristics of emotional inertia observed in individuals reporting depressive states (Costello, 1993; Bylsma et al., 2008; Kuppens et al., 2010; Rottenberg, 2017; Kahn et al., 2019).

Negative and positive reactivity has been observed to predict the risks for developing depression later. Multiple measures, like behavioral (Herres et al., 2016) and neural reactivity (Kujawa and Burkhouse, 2017; Stange et al., 2017) have been employed to investigate the role of emotional reactivity in processing affective stimuli among individuals with high and low risks for depression and reported mixed results. Behavioral studies (Abela et al., 2011; Hankin, 2012) have observed the increased emotional reactivity to negative stimuli and reduced emotional reactivity to positive stimuli in high-risk *versus* low-risk individuals. However, imaging studies have extended the support for blunted response to positive stimuli (Kujawa and Burkhouse, 2017) but reported contrasting results in the context of negative stimuli (Luking et al., 2016; Kujawa and Burkhouse, 2017). Studies favored mood-congruent or negative potentiation views when high compared to low risks individuals experience loss or sadness. Whereas when high-risk individuals were presented with threatening or negative images, they showed blunted response for both positive and negative stimuli (Kujawa and Burkhouse, 2017). Further, individuals reporting anhedonia or depressive mood state sadness have shown blunted PER but distinct NER. Unlike anhedonia, a depressed mood state favors negative information processing, supporting the mood-congruence view (Saxena et al., 2017; Bylsma, 2021).

In sum, the reduced PER to positive stimuli supports the low positive emotion model of depression (Clark et al., 1998) and extends its favor to the ECI model of depression (Bylsma et al., 2008; Rottenberg, 2017). Low positive emotional reactivity is reported as consistent (Bylsma et al., 2008; Abela et al., 2011; Hankin, 2012; Kujawa and Burkhouse, 2017; Bylsma, 2021) and a required screening method for depression (Davidson et al., 2002). However, the negative reactivity presents a conflict between mood-congruence and the ECI model of depression. The conflict could be explained by examining the difference in arousal levels of two negatively valenced stimuli (such as sadness and threatening stimuli), represented on different coordinate points of valence and arousal axis of the circumplex model of emotion (Bradley and Lang, 1994).

### Emotion and Concept Representation

Though the utility of investigating emotional reactivity in mood disorders or MDD is undeniable, it may not be sufficient for understanding depressive thought processes. It is important to note that if the depressive disorder is associated with affective and cognitive dysfunction alongside behavioral and physiological changes (Czerwińska and Pawlowski, 2019), then investigating the role of affective concepts representation would benefit in assessing emotional engagement and disengagement to both positive and negative stimuli.

How we code or represent and categorize concepts and store them in memory to retrieve later for survival has always been intriguing for cognitive science research. The recent development in the embodied and grounded theory of cognition (Borghi et al., 2018; Harpaintner et al., 2018) has enabled us to unravel the mystery of interaction between body and environment and how it shapes and constraints our cognitive abilities. According to such approaches, concrete concepts like a bottle, abstract concepts like freedom, and abstract affective concepts like love or despair are grounded in linguistic, social, emotional, and situational experiences alongside perception-action systems (Mazzuca et al., 2017; Borghi et al., 2018; Harpaintner et al., 2018). These concepts, once stored in memory, play a crucial role in various complex cognitive processes by tying our present interactions to our past experiences (Murphy, 2002; Barsalou, 2003).

According to embodied simulation theories, whenever the brain comes across a specific entity, the perceptual, motor and introspective states are stored in memory along with multimodal representations.

Later, in the absence of that entity, these representations help in simulating concepts. Studies have shown that these simulations are not fixed and change depending on the available context. Whenever a concept becomes active, it is never encoded independently but is supported by a meaningful situation in the background which is known as situated conceptualization (Barsalou, 2003, 2005, 2008; Barsalou et al., 2003; Barsalou and Wiemer-Hastings, 2005; Wilson-Mendenhall et al., 2010). A particular concept is constituted differently in every situation depending on the action, internal states, and perceptual construals. These theories point to the fact that the representation of concepts not only engage the sensorimotor resources but go one step further to depend on internal states and affective information [see Borghi et al. (2018) for review].

If an individual’s predisposition like mood state (Beck and Alford, 1967; Bower, 1981; Rottenberg et al., 2002; Bylsma et al., 2008) plays a critical role in processing affective information, it might influence the concept coding as well, especially affective concepts like joy, hope, and discouragement. Consistent with the mood-congruence argument of emotional reactivity, the associative network theory (Bower, 1981) emphasizes the role of mood in learning and memory. The associative links between the representation of different cognitive concepts (e.g., objects, events, or ideas), its affective state, and the mood state during the information acquisition and retrieval modulate the strength of learning and memory. However, studies (Wilson-Mendenhall et al., 2010; Borghi et al., 2018; Harpaintner et al., 2018; Kiefer and Harpaintner, 2020) examining the properties of emotional and non-emotional concepts lack analyzing the complex interplay between an individual’s mood state, stimulus’ affective nature, and the cognitive concepts. Since emotion has been observed as a critical component of concept formation or mental representation, the investigation of dysfunctional emotional state and its role in coding affective concepts appears quintessential for defining the heterogeneous features of depression.

### Present Study

We chose a sample representative of college-going or just graduated working adults, age range between 18 and 35 years old because of the prevalence and risks of depression during this stage of life. The overwhelming life-changing experiences of college or new jobs are, to name a few, determining factors that lead to the onset or development of depression at this age (Sagar et al., 2020; Sagar and Selvakumar, 2021). Given that emotional reactivity and representation depend not only on the stimuli’s affective nature but also on the individual mood state, it becomes imperative to examine the role of depressive symptoms in affective information processing and representation. The current study has two main objectives. First, examine the role of depression in emotional rating by employing a bi-dimensional self-assessment manikin (SAM) scale. Second, investigate the role of depression in affective concept representation by using the property generation task (see section “Property generation task”). We analyzed the emotional, semantic, and linguistic features generated in response to the emotional concepts.

Considering the ECI view of depression, we expect blunted emotional ratings and emotional properties to be generated by the depressed than a non-depressed group. However, if the negative mood facilitates negative information processing, then increased emotional ratings and negative features for negative stimuli (negative potentiation) are expected by the depressed than non-depressed. For positive stimuli, reduced positive ratings and positive features are expected by depressed than non-depressed group, supporting a positive attenuation view of depression.

## Materials and Methods

### Participants

Total hundred and eleven (53% female, 47% male, 0% other) English-speaking adults (Age: 18–30 years; Mean Age = 22.10 years; SD = 1.23 years) participated in the current study. The participation was totally based on voluntarily basis and no compensation was given to the participants. Participants were recruited via emails, Facebook, and Instagram advertisements. Quota selection sampling was used to recruit the participants. The inclusion criteria comprised Indian nationals, English speaking, age 18–30 years, education qualification with at least secondary education, and self and familial history of mental health.

Depression symptoms were assessed using the Beck Depression Inventory-II (Beck et al., 1996). The BDI-II is a widely used self-report inventory designed to measure the severity of depressive symptoms with acceptable internal consistency (around 0.9) and test-retest reliability (ranging from 0.73 to 0.96). Table 1 shows the distribution of the participants across various BDI categories. Based on previous research, the participants were divided into two groups (Beck and Alford, 1967; Moran et al., 2012). People scoring above 14 on the BDI scale were assigned to the depressed group and those scoring between 2 and 8 were assigned to the non-depressed group. Total six participants, scoring between 9 and 14 were excluded from the study since they belonged to the borderline and we wanted clear distinction between our two groups. Participants scoring 0 or 1 (*n* = 9) were also excluded from the study, since extremely low sores on BDI show positive mood state, extreme optimism and elevated social desirability (Clark et al., 1998). Kendall et al. (1987) also suggested that extremely low levels of depression symptoms may be associated with other forms of psychopathology. We also asked the participants whether they felt the need to consult a professional for mental health treatment and whether they ever took prescription for mental health treatment. 5 participants belonging to the non-depressed group and 26 belonging to the depressed group reported that they have consulted professional treatment and were excluded from our study. None of the participants in the two groups reported that they were under any medication for mental health. Moreover, no participants in the non-depressed group reported any history of familial mental illness whereas 4 participants out of 46 (8.69%) belonging to the depressed group reported history of familial mental illness. The final number of participants were 45 (19 females) in the non-depressed group and 20 (9 females) in the depressed group.

**TABLE 1:** Distribution of participants across BDI categories.

	Minimal	Mild	Moderate	Severe
BDI Range	(0–9)	(10–14)	(15–29)	(30–63)
Number of participants	59	6	42	4
Felt the need of consulting professional	5	2	22	4
Familial history of mental illness	0	0	3	1
Taken any psychiatric drug intervention	0	0	0	0
Effect on everyday experiences	0	0	0	0

In addition to BDI-II, participants also filled up a modified version of Perceived Stress Scale-10 related to COVID-19 [PSS-10-c; Campo-Arias et al. (2020)], which is a 10-item self-report inventory with an acceptable internal consistency (Cronbach’s alpha = 0.86), designed to measure the stress level of an individual in recent COVID times. The following Table 2 shows the means and standard deviations for each of the groups on age in years, BDI score and PSS-10-c scores.

**TABLE 2:** Participant demographics.

	Non-depressed (*n* = 45)		Depressed (*n* = 20)	
Variable	Mean	SD	Mean	SD
Age	22.44	1.19	22.6	1.44
**gender**				
Female	42*%*		45*%*	
Male	58*%*		55*%*	
**Educational qualification**				
senior high	6*%*		15*%*	
Graduates	81*%*		80*%*	
Post graduates	13*%*		5*%*	
BDI	5.22	2.03	18.14	6.39
PSS-10-C	15.64	3.29	18.75	3.78

### Stimuli

We used 15 emotional abstract words to investigate the differences in the ratings and the properties generated by the two population sets. These abstract words were a subset of the database introduced by Brysbaert et al. (2013) and were pre-categorized equally into five emotional categories: (1) high (i.e., positive)-valence high-arousal (HVHA) emotion words, (2) high-valence low-arousal (HVLA) emotion words, (3) low (i.e., negative)-valence high-arousal (LVHA) emotion words, (4) low-valence low-arousal (LVLA) emotion words and (5) neutral abstract (NAbs) words. We conducted a pilot study to choose a subset of emotional words from Brysbaert et al. (2013) to be used in this study. A convenient sample of 60 college students took part in this pretest to partially fulfill a course requirement. The individual predisposition emotional state of the entire sample was assessed using BDI-II (Beck et al., 1996) and the data of a subset of the sample (50 participants) showing minimal symptom ratings on the BDI-II scale were taken into consideration. This subset of the sample rated a total of 50 emotional words (10 belonging to each category) for valence and arousal. The age distribution in the pilot study resembled the age distribution in the main study (i.e., 18–30). The pilot study helped acquire the Indian affective experience for the given emotional stimuli. Based on these results, we selected a total of 15 words grouped into five categories. Each group was made such that it was geometrically most distant from other groups on the Valence-Arousal bi-dimensional scale using circumplex model (Bradley and Lang, 1994).

The final selection of words was comparable regarding concreteness as suggested by Brysbaert et al. (2013), word frequency (per million) as suggested by the Subtlex American Word Frequency norms (Brysbaert and New, 2009) and familiarity as suggested by the MRC familiarity norms (Coltheart, 1981). Average word frequency was 162.70 per-million words (SD = 280.329, range = 1.59–1114.98). Average word familiarity was 572.32 on a 100-700 point scale (SD = 31.22, range = 523–621). Average word concreteness was 2.27 on a 1–5 point scale (SD = 0.41, range = 1.75–3.04). All participants were given the same set of words to rate and generate properties.

### Tasks and Measures

Participants were asked to perform four tasks: (1) Affective Rating Task, (2) Concreteness Task, (3) Property Generation Task, (4) mental health surveys: BDI and PSS-c-10, followed by demographics questionnaires. For our analysis, we excluded the data from the concreteness rating task because it is out of the scope of the current study.

#### Affective Rating Task

Participants were instructed to report their feelings while viewing the presented word and rate it concerning valence (range: 1 = extremely unpleasant; 9 = extremely pleasant) and arousal (range: 1 = not at all arousing; 9 = extremely arousing) on a 9-point Likert scale via mouse click. We used the Self-Assessment Manikin scale (SAM), a non-verbal pictorial assessment technique (Bradley and Lang, 1994) to measure the self- reported valence and arousal ratings. The valence and arousal ratings (range: 1–9) for words belonging to the five stimuli categories (HVHA, HVLA, LVHA, LVLA, and NAbs) were averaged for every participant. Thus, giving us a single score for each stimuli category per participant.

#### Property Generation Task

Participants were instructed to write down thoughts, feelings, and/or memories that came into their minds for the presented emotional word. They were asked to write at least two grammatically sound sentences and avoid writing single words, phrases, or definitions. If no thought or feeling came to mind, the participants were told to skip that particular word. Participants were presented with 15 such words. Before starting the actual experiment, as a part of the instructions, subjects were shown an emotional word (which was not part of actual word stimuli) as an example, with its response written to familiarize themselves with the process.

The descriptive content created by the property generation task was analyzed using emotion and polarity analysis and semantic and linguistics feature analyses.

##### Emotion and Polarity Analysis

To measure the magnitude of different emotions (like joy, anger, sadness and optimism) present in the properties generated by the participants showing symptoms of depression and participants showing no or minimal symptoms, we took the help of the TweetEval model (Barbieri et al., 2020). Analogous to a human scoring the text generated by the participants on how much emotion is contained, the TweetEval model generated scores for each property on a scale from 0 to 1. Here, a higher score indicated a higher magnitude of a particular emotion present in the text. Similarly, another variant of the TweetEval model, capable of doing sentiment analysis, was used to generate polarity scores, which helped us quantify the positive and negative tone present in the text generated by the participants on a scale of 0-1 (higher the score, higher the magnitude of polarity). Each participant’s response was given to the model for generating emotion, and polarity scores were later averaged for each stimuli category, similar to the affective self-reported ratings.

##### Linguistic and Semantic Analysis

To analyze the semantic content present in the text given by the two population sets, we employed the use of linguistic inquiry and word count (LIWC) software (Stirman and Pennebaker, 2001). This text-analysis software gave us how much percentage of the data contained words belonged to a particular psychological dimension (affective words, motivational words, social words). Additionally, the program could give us the percentage of linguistic variables (like pronouns, adjectives, etc.) present in a text. Through this, we were able to do a contextual analysis of the text generated by our two sets of populations and see what differences exist between them. Like the emotion and polarity scores, participants’ responses were given to the LIWC software, which returned percentage scores for each dimension (range: 0–100%). These percentages were averaged across five stimuli categories, giving us a percentage score per category.

#### Mental Health Surveys

We asked participants to fill the standardized Beck’s depression inventory (BDI-II) (Beck and Alford, 1967) and perceived stress scale explicitly designed for COVID (PSS-c-10) (Campo-Arias et al., 2020). In these tasks, participants were instructed to read the presented statement carefully and respond to the choices that best describe their condition/state of mind. The BDI-II survey was scored by summing the ratings of its 21 items, where each item was rated on a 4-point scale ranging from 0 to 3. Similarly, the PSS-c-10 survey was scored by summing its ten items (range: 0–3).

### Procedure

**FIGURE 1:**
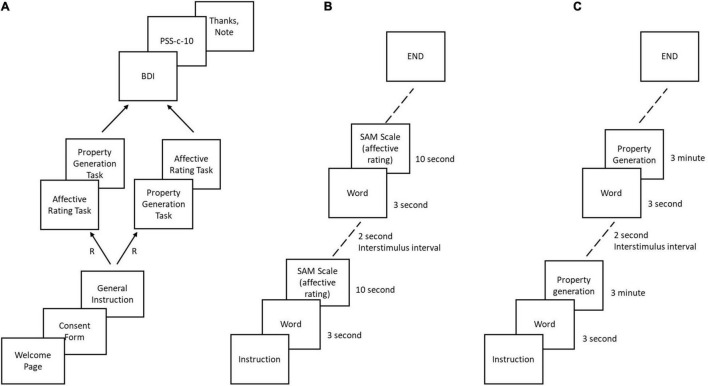
**(A)** Overall schematic flow of the experiment. R = random assignment **(B)** Schematic flow of affective rating task **(C)** Schematic flow of property generation task.

Participants completed the study via LabVanced (Goeke et al., 2017), a JavaScript web application developed for professional behavioral research. Participants were randomly assigned to the two tasks (affective rating and property generation task) (Figure 1A). The order of the two tasks was counterbalanced across participants. Half the participants performed the affective rating task first and the rest half performed the property generation task first in order. Each participant began the session with a welcome page, briefing about the purpose of the study, followed by the consent form, informing them about the anonymity and confidentiality of their data. All participants electronically gave their consent before starting the experiment. As participants progressed, they were informed about the task-specific instructions as per the task conditions (see section “Tasks and Measures” for more details). In both the tasks, we used fifteen words, three words each for the five emotional categories: HVHA, HVLA, LVHA, LVLA, and NAbs. We completely randomized the words displayed in both tasks to avoid any mood induction due to the affective nature of the stimuli. After completing the two main tasks, participants filled up a mental health questionnaire which consisted of BDI-II and PSS-10-c, and a demographic form. PSS-10-c was conducted in view of COVID-19 to assess the sense of stress possibly caused by COVID-19. The entire experiment took an average of 40 min to complete. The following Figure 1 depicts the schematic flow of the overall experimental session and the two main tasks.

## Results

### Participant Characteristics

Group comparisons were conducted using the Mann-Whitney U statistic since the data violated the Shapiro-Wilk’s normality test. The analysis did not yield significant age differences between the two groups (*p >* 0.1). However, the depressed group (*Mdn* = 19.5) were significantly higher in BDI ratings compared to the non-depressed group (*Mdn* = 5.5), *U* = 2300, *p <* 0.001, *r_*b*_* = 1.0, and PSS-10-c scores, *U* = 1806.5, *p <* 0.001, *r_*b*_* = 0.571. Results of the spearman’s correlation indicated a significant positive association between the BDI and PSS-10-c scores, *r_*s*_* = 0.518, *p <* 0.001. Moreover, no participants belonging to the non-depressed group reported any history of mental illness in their family, whereas 4 participants out of 46 (8.69%) belonging to the depressed group reported a history of mental illness in their family.

### Affective Rating-Valence and Arousal

The valence and arousal responses given by the participants in the affective rating task were analyzed using the Mann-Whitney U statistics for each stimulus category, since our data violated the Shapiro-Wilk’s normality test. To resolve the multiple testing correction, we used Bonferroni corrected p values to detect significance in our data. Studies show that Bonferroni correction is a conservative method to strictly avoid type-I error and control the family wise error-rate (Cabin and Mitchell, 2000; Armstrong, 2014). Since we ran multiple instances of the Mann Whitney U statistic, the original significance criterion (*p <* 0.05 and *p <* 0.01) was corrected to (*p_*bonf*_ <* 0.01 and *p_*bonf*_ <* 0.002) using the Bonferroni correction formula (Sedgwick, 2012). Table 3 reports the U statistic, median, and the Inter-Quartile Range (IQR) of valence and arousal values across each stimulus category. The median values of the depressed group are represented by *Mdn*_*D*_ and the non-depressed group are represented by *Mdn*_*ND*_.

**TABLE 3:** Affective rating on valence and arousal scale.

	Valence			Arousal		
	U	Mdn	IQR	U	Mdn	IQR
HVHA	263**			265**		
ND		8.66	0.66		8	1.33
D		7.66	1.5		5.83	2.66
HVLA	271.5**			347.5		
ND		8.00	1.00		5.66	3.33
D		7.5	1.83		5.16	3.41
LVHA	553.5			605.5		
ND		2	1.00		7.00	5.33
D		2.33	1.66		7.66	1.50
LVLA	527.5			598.5		
ND		1.66	1.33		5.00	1.33
D		2.00	1.08		4.67	4.75
NAbs	346			671.5		
ND		5.33	1.33		5.00	1
D		5	1.47		5.66	1.41

*Codes V = valence, A = arousal, H = High, L = Low, D = Depressed, and ND = Non-Depressed.*

**p_bonf_ ≤ 0.01.

For arousal ratings, the analysis indicated that the depressed group gave lower arousal compared to the non-depressed group for words belonging to HVHA. Moreover, the analysis also showed that the depressed group gave higher arousal compared to the non-depressed group for words belonging to the NAbs category. There was no significant differences in arousal ratings for HVLA, LVHA and LVLA categories. For valence ratings, the analysis showed that the depressed group gave lower valence rating compared to the non-depressed group for words belonging to HVHA, HVLA. There was no significant difference in valence ratings for LVHA and LVLA categories.

### Affective Representation

#### Emotion Content Analysis

The responses given by the participants in the property generation task were entered into the TweetEval model to obtain emotion scores for four emotions Anger, Joy, Sadness and Optimism. Table 4 reports the U statistic, median, and the Inter-Quartile Range (IQR) of these emotion scores. Since few portions of the data violated the assumption of normality, we conducted the non-parametric test of Mann-Whitney to analyze the differences between each emotion. To resolve the multiple testing correction, we used Bonferroni corrected p values to detect significance in our data. The original significance criterion (*p <* 0.05 and *p <* 0.01) was corrected to (*p_*bonf*_ <* 0.01 and *p_*bonf*_ <* 0.002) using the Bonferroni correction formula (Sedgwick, 2012). The median values of the depressed group are represented by *Mdn*_*D*_ and the non-depressed group are represented by *Mdn*_*ND*_.

**TABLE 4:** Emotion ratings calculated by TweetEval for stimuli categories.

	Anger			Joy			Optimism			Sadness		
	U	Mdn	IQR	U	Mdn	IQR	U	Mdn	IQR	U	Mdn	IQR
HVHA	327			392			324			521		
ND		0.03	0.04		0.54	0.26		0.29	0.29		0.05	0.06
D		0.02	0.01		0.51	0.33		0.18	0.15		0.05	0.18
HVLA	456			350			347			641.5**		
ND		0.04	0.03		0.43	0.28		0.32	0.34		0.18	0.14
D		0.04	0.06		0.35	0.18		0.24	0.13		0.26	0.25
LVHA	705***			567			227***			555		
ND		0.32	0.07		0.01	0.01		0.15	0.16		0.45	0.20
D		0.39	0.08		0.01	0.06		0.05	0.05		0.47	0.11
LVLA	405			512			241**			663**		
ND		0.05	0.1		0.01	0.006		0.18	0.15		0.69	0.18
D		0.03	0.04		0.01	0.03		0.05	0.12		0.83	0.16
NAbs	628			178.5***			416			592		
ND		0.11	0.1		0.18	0.09		0.23	0.19		0.41	0.18
D		0.18	0.14		0.05	0.14		0.22	0.18		0.45	0.14

*Codes V = valence, A = arousal, H = High, L = Low, D = Depressed, and ND = Non-Depressed.*

**p_bonf_ ≤ 0.01, ***p_bonf_ ≤ 0.001.

We examined the anger ratings across groups for each stimuli category with a Mann-Whitney test. The results indicated that the depressed group reported significantly higher levels of anger compared to the non-depressed group for the words belonging to the LVHA category. There were no significant differences between groups in terms of anger ratings for other stimuli categories. The examination of joy ratings revealed that the depressed group reported significantly lower levels of joy compared to the non-depressed group for the words belonging only to the NAbs category. There were no significant differences between groups in terms of joy ratings for the rest of the categories. For optimism ratings, the Mann-Whitney test showed that the depressed group reported significantly lower levels of optimism compared to the non-depressed groups for the words belonging to the LVHA and the LVLA category. There were no differences between groups for rest of the categories. For sadness ratings, the non parametric test revealed that the depressed group reported significantly higher levels of sadness compared to the non-depressed group for the words belonging to the HVLA, and the LVLA category.

#### Polarity Content Analysis

The responses given by the participants in the property generation task were entered into the TweetEval model to output positive and negative polarity scores. Table 5 reports the U statistic, median, and the Inter-Quartile Range (IQR) of these polarity scores. Due to the violation of the normality assumption, we conducted the Mann-Whitney U test to examine the polarity differences between the two groups. To resolve the multiple testing correction, we used Bonferroni corrected p values to detect significance in our data. The original significance criterion (*p <* 0.05 and *p <* 0.01) was corrected to (*p_*bonf*_ <* 0.01 and *p_*bonf*_ <* 0.002) using the Bonferroni correction formula (Sedgwick, 2012). The median values of the depressed group are represented by *Mdn*_*D*_ and the non-depressed group are represented by *Mdn*_*ND*_.

**TABLE 5:** Polarity ratings calculated by TweetEval for stimuli categories.

	Positive			Negative		
	U	Mdn	IQR	U	Mdn	IQR
HVHA	362.5			508.5		
ND		0.80	0.26		0.01	0.05
D		0.71	0.40		0.01	0.17
HVLA	359			635**		
ND		0.63	0.29		0.02	0.08
D		0.59	0.32		0.12	0.24
LVHA	264**			631**		
ND		0.04	0.11		0.63	0.23
D		0.02	0.05		0.73	0.28
LVLA	369			564		
ND		0.10	0.14		0.68	0.26
D		0.05	0.06		0.71	0.21
NAbs	209.5***			633**		
ND		0.30	0.15		0.36	0.21
D		0.13	0.18		0.48	0.23

*Codes V = valence, A = arousal, H = High, L = Low, D = Depressed, and ND = Non-Depressed.*

**p_bonf_ ≤ 0.01, ***p_bonf_ ≤ 0.001.

The Mann-Whitney test showed that the depressed group reported lower positive polarity scores compared to the non-depressed group for the words that belong to the LVHA, and the NAbs category. The Mann- Whitney test also showed that the depressed group reported higher negative polarity scores compared to the non-depressed group for the words that belong to the HVLA, LVHA, and the NAbs category.

#### Semantic Content Analysis

The responses given by the participants in the property generation task were entered into the LIWC program to output percentages of words belonging to three psychological processes affect, drives and social. Table 6 reports the U statistic, median, and the Inter-Quartile Range (IQR) for these processes. Due to the deviation of data from normality, we conducted the Mann-Whitney U test to examine the semantic differences between the participants showing depressive symptoms and participants showing no depressive symptoms. To resolve the multiple testing correction, we used Bonferroni corrected p values to detect significance in our data. The original significance criterion (*p <* 0.05 and *p <* 0.01) was corrected to (*p_*bonf*_ <* 0.01 and *p_*bonf*_ <* 0.002) using the Bonferroni correction formula (Sedgwick, 2012). The median values of the depressed group are represented by *Mdn*_*D*_ and the non-depressed group are represented by *Mdn*_*ND*_.

**TABLE 6:** LIWC percentages of psychological processes for stimuli categories.

	Affective			Motive			Social		
	U	Mdn	IQR	U	Mdn	IQR	U	Mdn	IQR
HVHA	378.5			352.5			362.5		
ND		15.22	6.14		10.87	6.08		14.44	9.24
D		13.03	7.99		10.53	6.11		12.07	7.72
HVLA	469.5			269**			322		
ND		11.54	6.62		8.05	5.90		7.59	7.07
D		14.12	7.08		4.96	4.14		5.51	3.76
LVHA	246.5**			318			274**		
ND		14.63	5.71		9.30	6.20		7.44	6
D		9.91	5.68		5.85	6.01		4.23	4.02
LVLA	355.5**			257**			344.5		
ND		15.15	8.70		9.68	5.85		9.68	9.25
D		10.21	8.96		7.20	2.50		6.09	6.73
NAbs	364			334			430		
ND		10.20	5.13		11.11	5.67		6.86	7.06
D		8.35	3.48		9.82	3.42		6.23	7.11

*Codes V = valence, A = arousal, H = High, L = Low, D = Depressed, and ND = Non-Depressed.*

**p_bonf_ ≤ 0.01.

The Mann-Whitney test revealed that the depressed group used less percentage of affective words in their properties generated compared to the non-depressed group for words belonging to the LVHA, and the LVLA category. The Mann-Whitney test revealed that the depressed group used less percentage of motivational words (drives) in their properties generated compared to the non-depressed group for words belonging to the HVLA, and the LVLA category. For social words, the Mann-Whitney test revealed significant differences between the depressed group and the non-depressed group for LVHA category.

#### Linguistic Content Analysis

Linguistic inquiry and word count (LIWC) was used to obtain the percentages of different linguistic variables first-person singular pronouns, first-person plural pronouns, adjectives and negation present in the properties generated by the participants. Table 7 reports the U statistic, median, and the Inter-Quartile Range (IQR) for these variables. We conducted a Mann-Whitney U test to analyze the linguistic differences that exist between the two groups. To resolve the multiple testing correction, we used Bonferroni corrected p values to detect significance in our data. The original significance criterion (*p <* 0.05 and *p <* 0.01) was corrected to (*p_*bonf*_ <* 0.01 and *p_*bonf*_ <* 0.002) using the Bonferroni correction formula (Sedgwick, 2012). The median values of the depressed group are represented by *Mdn*_*D*_ and the non-depressed group are represented by *Mdn*_*ND*_.

**TABLE 7:** LIWC percentages of linguistic variables for stimuli categories.

	I			We			Adjective			Negation		
	U	Mdn	IQR	U	Mdn	IQR	U	Mdn	IQR	U	Mdn	IQR
HVHA	633.5**			401.5			430.5			439.5		
ND		6.38	11.43		0.96	1.33		6.80	4.63		0.00	2.13
D		13.11	7.56		0.60	0.00		6.79	4.50		0.00	1.82
HVLA	489.5			341.5			427.5			567		
ND		6.78	8.21		0.85	0.00		10.30	4.69		0.00	1.89
D		7.98	7.14		0.24	0.00		9.64	5.70		1.63	3.18
LVHA	634.5**			333.5			265**			607		
ND		7.69	7.61		1.01	2.04		9.03	3.89		2.17	2.86
D		11.32	6.04		0.24	0.00		6.64	4.36		3.39	1.37
LVLA	599			401			328.5			555		
ND		7.69	8.31		0.85	1.11		5.95	4.33		3.23	2.55
D		11.70	10.50		0.52	0.00		4.47	4.35		3.96	3.06
NAbs	522.5			400.5			310.5			506.5		
ND		8.82	12.58		1.20	1.05		4.31	3.06		1.79	2.7
D		11.31	7.52		0.52	0.00		2.68	3.19		2.41	3.25s

*Codes V = valence, A = arousal, H = High, L = Low, D = Depressed, and ND = Non-Depressed.*

**p_bonf_ ≤ 0.01.

In case of first person singular pronouns (e.g., “I”), the Mann-Whitney test showed that the depressed group used a higher percentage compared to the non-depressed group for words that belong to the LVHA. There were no significant differences between groups for rest of the categories. For adjectives, the analysis revealed that the depressed group used lower percentage of adjectives compared to non-depressed group for words belonging to the LVHA category. There were no differences between groups for rest of the categories. In the case of first-person plural pronouns (e.g., “we”) and negation, the Mann-Whitney test did not reveal any significant differences between the groups.

## Discussion

The current study examined the relationship between depressive symptoms and emotion experience and representation. We showed emotional stimuli of varying hedonic and arousal content to individuals who reported depressive symptoms and no-depressive symptoms or healthy control. Participants were asked to record their affective experiences using the SAM scale and describe their feelings after reading those concepts using the property generation task. These descriptive typed responses were analyzed on multiple levels to understand how the two respective groups interpret and represent emotional concepts comprehensively. We will first discuss the role of depressive symptoms in emotional reactivity, followed by depressive symptoms in conceptualizing the affective concepts. The representation of affective concepts is analyzed as emotional content analysis alongside semantic and linguistic properties while thinking about the emotionally evocative concepts.

### Emotional Reactivity

Emotional reactivity was measured as a positive or negative valence and high and low arousal response to the corresponding emotionally valenced stimulus. We conducted a comparative analysis between the non- depressed and depressed groups to examine the role of depression in emotional reactions reported against affective stimuli. We observed a significant difference between the non-depressed and depressed groups for positive reactivity, but no difference was observed for negative reactivity. Participants reporting depressive symptoms (BDI scores greater than 14) showed reduced valence ratings for the highly positive words with high and low arousal levels. The reduced positive reactivity by the depressed group in comparison to the non-depressed group supports the positive attenuation theory, suggesting a weak experience of pleasant feeling.

The lack of significant difference between the non-depressed and depressed groups’ emotional reaction to the negative stimuli fails to support the assumption of biased cognitive processing for unpleasant stimuli during the depressive state of mind. Unlike previous findings (Beck and Alford, 1967; Bower, 1981; Bylsma et al., 2008), the current results neither favor the assumption of negative potentiation or mood-congruence (Beck and Alford, 1967), nor showed any support for negative attenuation as a part of integrated ECI view of depression (Bylsma et al., 2008). Participants with depressive symptoms showed neither increase (Beck and Alford, 1967; Abela et al., 2011; Hankin, 2012) nor decrease (Bylsma et al., 2008; Saxena et al., 2017; Bylsma, 2021) in emotional reactivity to the words expected to evoke negative experience.

Although an unpleasant affective rating did not show any clear directions on negative reactivity, the current results with an emotional response to affective stimuli demonstrate no substantial support for the ECI assumptions. According to ECI (Bylsma et al., 2008), patients with depressive symptoms exhibit a weakened response to all emotional cues, regardless of the valence of the words. The depressive symptoms are associated with negative thought processes and lead to general disengagement and reduced motivated activities, suggesting economic use of cognitive resources to process any information independent of their emotional context (Bylsma et al., 2008). Considering ECI assumptions (Bylsma et al., 2008), the response to the negative stimuli could have been pushed toward neutral or moved away from the extreme negative experiences because of the lack of motivation to respond. However, the current result does not show a significant shift toward neutral even when the presented words matched the extreme negative categories (Brysbaert and New, 2009).

On the other hand, the absence of negative potentiation could be explained by the matched extreme negatively valenced stimuli. The negative ratings of the chosen stimuli were extreme, ranging between 1.5 to 2.5 affective ratings, leaving not much room for more extreme responses and thus resulting in a lack of negative potentiation. In addition, we used negative words that were expected to evoke low arousal negative response (e.g., sadness, LVLA) and high arousal negative response (e.g., fear, LVHA). However, unlike previous studies, we did not observe a differential response for LVHA and LVLA words. While comparing MDD and healthy control, previous studies have shown negative potentiation with sadness (i.e., representing LVLA) (Kujawa and Burkhouse, 2017) and blunted response with fear or threatening stimuli (i.e., representing LVHA) (Kujawa and Burkhouse, 2017). The differences in the participant profile (clinical *versus* no clinical diagnosis of depressive symptoms) and modality of observations (e.g., behavioral, physiological, or imaging) could explain such differences and the lack thereof.

In sum, using the SAM scale as a measurement, the current study does not support either of the two competing theoretical assumptions, the ECI (Bylsma et al., 2008) and negative potentiation or mood- congruence (Beck and Alford, 1967; Beck et al., 1979; Bower, 1981). However, the lack of support for mood-congruence could be interpreted with a caveat of the type of stimuli that have been used for this study. Our result fails to dismiss the possibilities of mood-congruence because of the highly negative valenced stimuli and raises concerns about processing negative stimuli with varying valence ratings.

Alongside valence, we also used words with varying arousal to measure emotional reactivity to high compared to low arousing stimuli. We compared the self-reported arousal rating between non-depressed and depressed group and extended the testing of positive attenuation and two competing theories, i.e., mood congruence theory (Beck and Alford, 1967; Bower, 1981) and ECI (Bylsma et al., 2008), for emotional reactivity to understand depression. We observed that participants showing symptoms of depression compared to participants showing no symptoms of depression reported significantly low arousal for high arousing positive words, i.e., HVHA. However, unlike positive emotional reactivity for pleasantly valenced stimuli, the positive emotional reactivity for arousal rating was specific for high arousing pleasant stimuli (i.e., HVHA). No significant difference was observed for low arousing pleasant words (i.e., HVLA). Emotional reactivity for arousal rating fails to support previous views on depression, namely positive attenuation, ECI, and mood-congruence.

Further, the current result focusing on response to arousal presents a contrast to the under-arousal model of depression (Grossberg, 1972). The under-arousal model posits that individuals with depressive symptoms exhibit reduced arousal response for all categories of emotion and explain the aberration in psychomotor activities. Only a few studies (Buyukdura et al., 2011; Benning and Ait Oumeziane, 2017) have used arousal as a measure of psychomotor assessment for understanding depression, using the under-arousal model of depression. Our results support the under-arousal model of depression for positive stimuli with high arousal but fail to extend the support for other emotionally evocative stimuli. No difference between depressed and non-depressed individuals’ arousal experience for negative stimuli suggests that individuals with self-report symptoms of depression or propensity to depression may differ from MDD patients’ experience of arousal. More systematic studies are required to evaluate the role of arousal in the emotional processing of people showing depressive symptoms and MDD patients compared to a non-depressed group.

In the context of emotional reactivity, the current results do not provide strong support for any of the two major competing theories, i.e., ECI (Bylsma et al., 2008) and mood congruence (Beck and Alford, 1967; Bower, 1981). However, we observed strong and unanimous support for positive attenuation theory (Bylsma et al., 2008) for positively valenced stimuli. In sum, individuals with self-reported depressive symptoms *versus* no depressive symptoms, using BDI, showed a significantly reduced emotional reactivity for pleasant words regardless of their arousal levels and reduced arousal reactivity specific for extremely positive words with high arousal.

### Emotional Concept Representation

#### Emotion and Polarity Differences

In addition to emotional reactivity, we examined the role of depression in mental representation. We wanted to understand how an individual with depressive *versus* no depressive state differ in their thinking for affective words. We used computation models to analyze the descriptive features of affective words generated by the participants during the property generation task. The comparative analyses between the non-depressed and depressed groups for sentiment and semantic analyses helped us learn about an individual’s thinking style with depressive and no-depressive thought processes. The affective concept feature analyses enabled us to examine the consistency between emotional reaction and emotional content representation reported by the individuals.

Our study attempted to evaluate the magnitude of discrete emotion (anger, joy, optimism, sadness) individuals of different mental states display when presented with emotional stimuli. Our results suggest that individuals belonging to the depressed group show higher anger levels than individuals belonging to the non-depressed group while responding to negative stimuli of high arousal. Moreover, we also observe that the depressed group reports significantly lower joy content while responding to neutral stimuli. We further observed that the depressed group showed lower optimism toward negative stimuli and increased sadness for low arousing stimuli regardless of their valence levels. The current results support the notion that individuals with depressive symptoms show pessimistic behavior, with increased sadness and decreased optimistic feeling (Chang and Sanna, 2001; Hart et al., 2008).

In addition to emotion analysis, we also attempted to measure the polarity of properties generated by the two population sets. Participants with self-reported depressive symptoms showed higher negative polarity for negative stimuli of high arousal and positive stimuli of low arousal, and lower positive polarity selectively for neutral and negative stimuli of high arousal level. These findings partially support the notion that depressed individuals tend to focus on the negative aspects of life and thus use more negative words in their properties (Beck and Alford, 1967; Leis et al., 2019).

It is important to note that emotion and polarity analyses helped us understand the thinking style of an individual suffering from depressive thought processes than otherwise, similar to the studies of emotional reactivity. We chose the paradigm that could simulate the features of autobiographical narratives in a controlled manner and help us analyze an individual’s thinking style with a depressive and no-depressive state of mind. The paradigm of our study was based on self-rumination, where participants were told to write their thoughts toward emotionally evocative words. In this way, the emotional words acted as biographical stimuli. Since language and cognition go hand in hand (Perlovsky and Sakai, 2014; Winskel and Luksaneeyanawin, 2014), the written response by participants seems to be a better way to understand how individuals process emotional concepts compared to discrete and quantitative reporting of affective state (like Likert scale). The notion behind it was that we write how we think, especially when we were asked to write as first thoughts.

The emotion content and polarity analyses enabled a better understanding of the emotional reactivity data. We observed contrast between positive emotion reactivity response and positive content and polarity result. Though reported lower arousal and positive emotion reactivity to pleasant stimuli, we observed no difference between the depressed and non-depressed groups for positive polarity content generated for pleasant stimuli regardless of their arousal level. The positive content, joy and optimism did not show consistency with attenuated response to pleasant response from depressed compared to the non-depressed group. However, descriptive content from depressed group showed low positive polarity for unpleasant words with low arousal level and used low optimistic content for unpleasant words regardless of their arousal level. We further observed an inconsistent result when comparing the negative content and polarity responses to the negative emotional reactivity response. We observed high negative polarity for unpleasant stimuli with high arousal levels, high sadness content for unpleasant stimuli with low arousal, and high anger content for unpleasant stimuli with high arousal.

As argued earlier, the lack of mood-congruent emotional reactivity could be because of the highly negative stimuli used in the current study. Therefore, individuals with depressive mood states could not register more extreme responses for the corresponding negative stimuli. However, when individuals with a depressive mood state were asked to describe their feeling associated with the affective concept, they could deliberate on the content of the emotional experience while reading those words. The psychological processes required to perform the two tasks could explain the inconsistency between the emotional reactivity and emotional descriptive content analyses.

Unlike emotional reactivity, the descriptive content allowed us to examine their knowledge representation of a given affective concept. The match between the affective concept and individual negative schema (negative core belief, e.g., I am useless) determines the associative strength between the nodes/concepts used to describe their feeling (Bower, 1981). The depressed individuals engage in more negative thinking, called depressive brooding (Joormann et al., 2006), and show poor executive control in shifting focus to alternative views (Derakshan and Eysenck, 2010), which strengthens the negative schema. Upon describing when asked, the process may activate the most available negative schema, resulting in mood-congruent descriptive content (Beck and Alford, 1967; Bower, 1981).

### Semantic and Linguistic Representation

We analyzed the semantic and linguistic components of the properties generated by the participants to understand the interpretation of emotional concepts. Analyzing the text generated, we observed that the healthy non-depressed used a higher percentage of affective words than individuals with depressive symptoms while responding to negative stimuli of high arousal. This means that even though people showing depressive symptoms react stronger and use more anger words, they do not generally use affective words. Moreover, we also observe that the individuals belonging to the depressed group use fewer motivational words while generating properties for low arousing stimuli regardless of their valence. A characteristic feature of depression is the diminishing of motivation. Cognitive impairments due to depression are linked closely to impairments in motivational processes (Crocker et al., 2013; Grahek et al., 2019). Grahek et al. (2019) proposed a framework in which the biases in cognition in depressed populations arise from the variations in important components of motivation like reward and anticipation. Social integration/disengagement theories (Durkheim, 1897/1951), talk about how individuals suffering from depression are less socially engaged and have more dysfunctional social relationships (Elmer and Stadtfeld, 2020), which is explained by our data as well. Participants showing depressive symptoms used less social words for negative stimuli of high arousal compared to participants showing minimal depressive symptoms.

In this study, we also compared certain linguistic variables focusing on self used by people showing depressive symptoms and people showing minimal or no depressive symptoms. The findings were consistent with previous studies (Bucci and Freedman, 1981; Pyszczynski and Greenberg, 1987; Stirman and Pennebaker, 2001; Rude et al., 2004; Tausczik and Pennebaker, 2010; Leis et al., 2019) that depressed individuals used first-person singular pronouns more frequently than healthy control. This increase indicates that depressed individuals tend to interpret situations in terms of self-focus. Our findings that the depressed group used a fewer number of adjectives than the non-depressed group was consistent with (Leis et al., 2019). However, we could not find evidence that depressed individuals used a fewer number of first-person plural pronouns and negative words.

## Conclusion and Future Directions

The current results provide insight about the non-clinically diagnosed population, who have self-reported the symptoms of depression and have reported how they feel and think about affective words. The differential affective response and conceptual representation suggest vulnerability to or risks of depression in future with a caveat of no comparison between individuals with self-reported symptoms of depression and clinically diagnosed unipolar depression or major depressive disorder.

We observed mixed responses supporting differing views of depression. The emotional reactivity supported the reduced positive emotion reactivity unanimously for valence rating (Bylsma et al., 2008). However, the emotional content analyses suggest no clear support for competing views of depression (Beck and Alford, 1967; Bower, 1981; Rottenberg et al., 2002; Bylsma et al., 2008). We observed increased sadness for low arousing stimuli regardless of their valence and selective inclination toward negative polarity for pleasant stimuli with low arousal and unpleasant stimuli with high arousal. In addition, the current data did not support the under arousal model of depression (Gross and Levenson, 1993; Benning and Ait Oumeziane, 2017). Positive emotional processing of the self-reported depressive group aligns with clinical unipolar depression or major depressive disorder emotional processing. However, the negative emotional processing, especially the comparison between emotional reactivity and mental representation, demands further examination between cohorts of self-reported depressive symptoms, no-symptoms, and MDD patients.

The current emotional content and polarity analyses and their contrast with emotional reactivity suggest a systematic evaluation of affective response against emotionally evocative stimuli while considering the individual mood state. Given the importance of emotional reactivity in depression risk assessment, the contrasting results with emotional content and emotional polarity indicate the importance of multi- dimensional and multi-modal risk assessment for effective intervention plans.

## Data Availability Statement

The raw data supporting the conclusions of this article will be made available by the authors, without undue reservation.

## Ethics Statement

The studies involving human participants were reviewed and approved by Institute Review Board Committee, International Institute of Information Technology, Hyderabad, India. The patients/participants provided their written informed consent to participate in this study.

## Author Contributions

HS and PS conceptualized this manuscript. HS designed and conducted the experiment, performed the data analysis, and wrote the first draft. PS conceived the original idea, discussed the theoretical motivation with HS, and advised HS at every stage of the project, including theoretical relevance, stimuli selection, experiment design, data collection, data analysis, result interpretation, and multiple drafts. Both authors collaborated on the project’s primary focus, and worked together on critical revisions of the manuscript.

## Conflict of Interest

The authors declare that the research was conducted in the absence of any commercial or financial relationships that could be construed as a potential conflict of interest.

## Publisher’s Note

All claims expressed in this article are solely those of the authors and do not necessarily represent those of their affiliated organizations, or those of the publisher, the editors and the reviewers. Any product that may be evaluated in this article, or claim that may be made by its manufacturer, is not guaranteed or endorsed by the publisher.
